# Muscle-Related Polymorphisms (*MSTN* rs1805086 and *ACTN3* rs1815739) Are Not Associated with Exceptional Longevity in Japanese Centenarians

**DOI:** 10.1371/journal.pone.0166605

**Published:** 2016-11-18

**Authors:** Noriyuki Fuku, Rafael Alis, Thomas Yvert, Hirofumi Zempo, Hisashi Naito, Yukiko Abe, Yasumichi Arai, Haruka Murakami, Motohiko Miyachi, Helios Pareja-Galeano, Enzo Emanuele, Nobuyoshi Hirose, Alejandro Lucia

**Affiliations:** 1 Graduate School of Health and Sports Science, Juntendo University, Chiba, Japan; 2 School of Medicine and Research Institute “Dr. Viña Giner”, Molecular and Mitochondrial Medicine, Catholic University of Valencia San Vicente Mártir, Valencia, Spain; 3 Servicio de Nefrología, Hospital Universitario y Politécnico La Fe, Valencia, Spain; 4 School of Doctorate Studies and Research, Universidad Europea de Madrid, Madrid, Spain; 5 Center for Supercentenarian Study, Keio University School of Medicine, Tokyo, Japan; 6 Department of Health Promotion and Exercise, National Institute of Health Nutrition, Tokyo, Japan; 7 European University of Madrid, Madrid, Spain; 8 Research Institute of Hospital 12 de Octubre (“i+12”), Madrid, Spain; 9 2E Science, Robbio, Pavia, Italy; Yale School of Public Health, UNITED STATES

## Abstract

Myostatin (*MSTN*) and α-actinin-3 (*ACTN3*) genes are potentially associated with preservation of muscle mass and oxidative capacity, respectively. To explore the possible role of these genes in exceptional longevity (EL), the allele/genotype frequency distribution of two polymorphisms in *MSTN* (rs1805086, K153R) and *ACTN3* (rs1815739, R577X) was studied in Japanese centenarians of both sexes (n = 742) and healthy controls (n = 814). The rs1805086 R-allele (theoretically associated with muscle mass preservation at the expense of oxidative capacity) was virtually absent in the two groups, where genotype distributions were virtually identical. Likewise, no differences in allele (*p* = 0.838 (women); *p* = 0.193 (men); *p* = 0.587 (both sexes)) or genotype distribution were found between groups for *ACTN3* rs1815739 (*p* = 0.975 (women), *p* = 0.136 (men), *p* = 0.752 (both sexes)). Of note, however, the frequency of the rs1805086 R-allele observed here is the lowest been reported to date whereas that of the ‘highly oxidative/efficient’ rs1815739 XX genotype in Japanese male centenarians (33.3%) or supercentenarians of both sexes (≥110 years) are the highest (32.6%), for a non-American population. No definite conclusions can be inferred in relation to EL owing to its lack of association with both rs1815739 and rs1805086. However, it cannot be excluded that these gene variants could eventually be related to a “healthy” metabolic phenotype in the Japanese population. Further research might determine if such metabolic profile is among the factors that can potentially predispose these individuals to live longer than Caucasians and what genetic variants might be actually involved.

## Introduction

As life expectancy in developed countries rises, the number of elderly people and among them those showing exceptional longevity (EL, ≥100 years) steadily climbs. A major problem associated with ageing is the gradual decline in those systems and organs that determine physical fitness, notably the skeletal muscle tissue. Accelerated age loss of muscle strength (sarcopenia) is associated with higher mortality risk and genes potentially associated with preservation of muscle mass and metabolic function could be potentially associated not only with healthy ageing and disability risk, but also with EL [1].

One candidate to modulate muscle mass during aging is the gene encoding myostatin (*MSTN*, also termed ‘growth differentiation factor 8’; MIM#601788). Myostatin is a highly conserved member of the transforming growth factor-β superfamily that is expressed predominantly in the muscle tissue and mechanistic studies have revealed its role as a negative modulator of muscle mass [2, 3], with over-expression of this molecule being involved in the development of cachexia in cancer patients [4]. Besides its potential role in the modulation of sarcopenia and muscle metabolism, the rs1805086 (2379A>G) polymorphism in exon 2 of *MSTN* gene, which causes a Lys(K)153Arg(R) substitution, has been linked with a higher likelihood of reaching EL in North-Italian and Spanish cohorts [5].

Progressive mitochondrial dysfunction notably affecting the nervous and muscle tissue, *e*.*g*., due to accumulation of deletions in mitochondrial DNA (mtDNA), is a major hallmark of the aging process [6] and thus genetic variants associated with muscle oxidative metabolism could also potentially influence EL. One such candidate is the Arg(R)577Ter(X) (rs1815739) polymorphism in the gene (*ACTN3*, MIM#102574) encoding α-actinin-3, a sarcomeric protein expressed in skeletal-glycolytic fibers [7], which can potentially affect not only exercise performance [8], but also health-related phenotypes owing to its influence on muscle oxidative metabolism. In this regard, classic studies in artificially selected rats showed that improved oxidative pathways in muscle mitochondria may be a common factor linking physical fitness with decreased disease risk and higher survival [9].

Japan has the longest life expectancy worldwide, as well as the highest number of centenarians. This population represents an interesting model to investigate the genetic factors involved in EL. We aimed to study the potential role of *MSTN* and *ACTN3* genes in EL by analyzing the genotype/allele frequency distribution of *MSTN* rs1805086 and *ACTN3* rs1815739 in a large cohort of Japanese centenarians. We also studied a control group of younger adults, and sex was taken into account in the statistical analyses.

## Material and Methods

The study was approved by the local ethics Committee (Keio University and National Institute of Health and Nutrition, Japan) and written consent was obtained from all the subjects. Seventy hundred and forty-two centenarians (100–116 years, 623 women) and 814 healthy controls (23–65 years, 601 women), all from the same Asian (Japanese) descent were studied. The centenarians were recruited from two previously described [10] prospective cohorts: The Tokyo Centenarians Study (TCS) and the Semi-Supercentenarians Study in Japan (SSC-J). The TCS cohort includes 304 centenarians randomly selected between July 2000 and May 2002 among those living in the 23 wards of metropolitan Tokyo (representing 17.5% of an estimated 1735 centenarians living in this area in the aforementioned period) [10]. The SSC-J is a nationwide longitudinal survey consisting mainly of individuals aged 105 years or older, which started in 2002 and had a total of sample size of n = 450 by end of November 2011. The prevalence rates of hypertension, coronary artery disease and dementia in the Japanese centenarians were 63.6%, 28.8% and 59.4%, respectively [11]. Inclusion criteria for the control group, which was recruited during 2007–2012 from people participating in a Nutrition and EXercise Intervention Study (NEXIS, registered at ClinicalTrials.gov, Identifier: NCT00926744), were being a man or woman aged 23–65 years without a history of stroke, cardiovascular disease, chronic renal failure, or walking difficulties related to knee or back pain [12]. People with the aforementioned conditions were excluded from NEXIS because their exercise habits could be partly influenced by their disease or pain.

Total DNA was isolated from venous blood by use of QIAamp DNA Blood Maxi and/or Mini Kit (QIAGEN, Hilden, Germany). The rs1805086 and rs1815739 polymorphisms were genotyped using TaqMan SNP genotyping assays (assay ID, C____282184_30 and C____590093_1_, respectively) and a real-time thermocycler (LightCycler 480, Roche Applied Science, Mannheim, Germany). A total of 5 μL of genotyping mixture containing 2.5 μL of GTXpress^TM^ Master Mix, 0.125 μL of assay mix (40x), and 1.375 μL of distilled water was mixed with 1 μL of genomic DNA (10 ng/μL) in each reaction. PCR 384-well plates were read on the thermocycler using the end-point analysis mode. Allelic discrimination analysis was performed with a LightCycler 480 SW software version 1.5.1.62 (Roche Applied Science, Mannheim, Germany). Four or five negative controls were included on each plate.

Genotype and allele frequencies were compared between groups using the χ^2^ and the Fisher exact test (α set at 0.05).

## Results and Discussion

**Fig 1:**
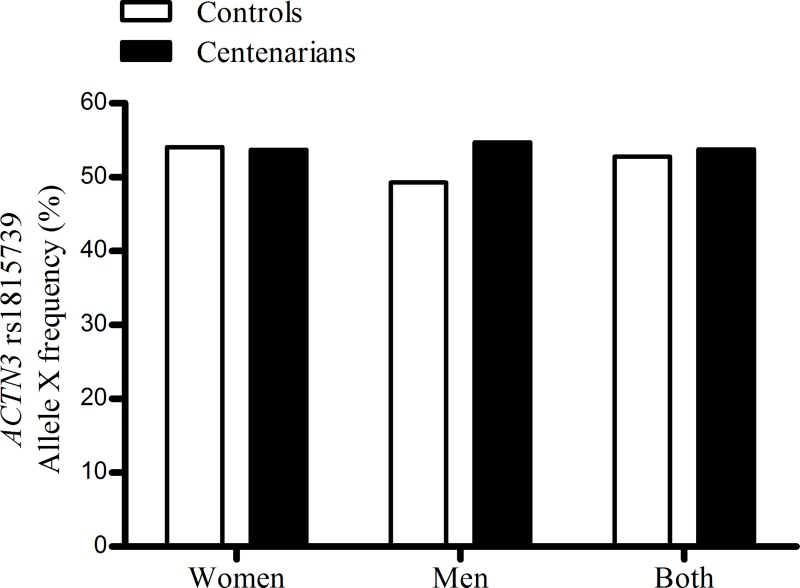
Allele frequency in the R577X (rs1815739) variation in the α-actinin-3 (*ACTN3*) gene in controls and centenarians. No significant differences were found between groups (Fisher’s test: *p* = 0.838 (women); *p* = 0.193 (men); *p* = 0.587 (both sexes)).

The *MSTN* rs1805086 K-allele was highly predominant in the whole cohort and genotype distribution was virtually identical in centenarians and controls (**Table 1**). The *ACTN3* rs1815739 genotype distribution did not differ between the two groups regardless of sex or model of analysis (co-dominant or dominant, **Table 1**), and no differences were found in allele frequency either (**Fig 1**).

**Table 1:** Genotype distributions of the myostatin (*MSTN*) rs1805086 and α-actinin-3 (*ACTN3*) rs1815739 variations in Japanese centenarians and controls.

***MSTN***		Women					Men					All together				
**K153R (rs1805086)**		Centenarians(n = 616)		Controls (n = 601)			Centenarians (n = 117)		Controls (n = 213)			Centenarians (n = 733)		Controls (n = 814)		
		n	%	n	%		n	%	n	%		n	%	n	%	
Codom	KK	615	99.8	601	100.0		117	100.0	213	100.0		732	99.9	814	100.0	
	KR	1	0.2	0	0.0		0	0.0	0	0.0		1	0.1	0	0.0	
	RR	0	0.0	0	0.0		0	0.0	0	0.0		0	0.0	0	0.0	
		n	%	n	%		n	%	n	%		n	%	n	%	
Dom	KK/KR	615	99.8	601	100.0		117	100.0	213	100.0		732	99.9	814	100.0	
	RR	1	0.2	0	0.0		0	0.0		0.0		1	0.1	0	0.0	
***ACTN3***		Women					Men					All together				
**R577X (rs1815739)**		Centenarians (n = 612)		Controls (n = 596)			Centenarians (n = 117)		Controls (n = 213)			Centenarians (n = 729)		Controls (n = 809)		
		n	%	n	%	χ^2^ *p*-value	n	%	n	%	χ^2^ *p*-value	n	%	n	%	χ^2^ *p*-value
Codom	RR	124	20.3	118	19.8	0.975	28	23.9	53	24.9	0.136	152	20.9	171	21.1	0.752
	RX	319	52.1	311	52.2		50	42.7	110	51.6		369	50.6	421	52.0	
	XX	169	27.6	167	28.0		39	33.3	50	23.5		208	28.5	217	26.8	
		n	%	n	%	Fisher’s *p*-value	n	%	n	%	Fisher’s *p*-value	n	%	n	%	Fisher’s *p*-value
Dom	RR/RX	429	72.0	443	72.4	0.898	78	66.7	163	76.5	0.069	521	71.5	592	73.2	0.458
	XX	167	28.0	169	27.6		39	33.3	50	23.5		208	28.5	217	26.8	

The failure rate of genotyping for *MSTN* rs1805086 was 1.2% in the centenarians’ group and 0.0% in controls (0.6% overall). For *ACTN3* rs1815739 the genotyping failure rates were 1.8% and 0.6% in centenarians and controls, respectively (1.2% overall). Due to the results for *MSTN* rs1805086 (see below) the Hardy-Weinberg equilibrium (HWE) was not tested. For the *ACTN3* rs1815739, HWE was observed in controls (*p* = 0.209) and centenarians (*p* = 0.620). Abbreviations: Codom, co-dominant; Dom, dominant.

Although no insight on the potential role of the *MSTN* rs1805086 variant on EL can be extrapolated from our data, the highly homogeneous genotype found in the Japanese population is somehow striking. This polymorphism has been previously analyzed in other cohorts of a different ethnic/geographic origin, which showed a higher prevalence of the variant R-allele (**Table 2**). Interestingly, populations from the African continent (which conversely are those with a lower prevalence of the variant *ACTN3* X-allele–see below) show the highest prevalence of the mutant R-allele (**Table 2**).

**Table 2:** Allele frequencies in the current study versus those previously reported in the literature for the K153R (rs1805086) variation in the myostatin (*MSTN*) gene.

Cohort	n	K-allele (%)	R-allele (%)	Reference
Japanese Controls	812	100%	0%	Present study
Japanese Centenarians	733	99.9%	0.1%	
Chinese Han men	94	93.6%	6.4%	(Li et al. 2014) [14]
Caucasian Americans	95	96.3%	3.7%	(Ferrell et al. 1999) [22]
African Americans	93	83.9%	16.1%	
Spanish controls	384	97.3%	2.7%	(Garatachea et al. 2013) [5]
Spanish centenarians	156	92.3%	7.7%	
Italian controls	316	97.0%	3.0%	(Garatachea et al. 2013) [5]
Italian centenarians	79	92.4%	7.6%	
Elder Turkish	152	98.0%	2.0%	(Tosun Tasar et al. 2015) [23]
Spanish (*Alpujarra*)	70	92.0%	7.1%	(Fernandez-Santander et al. 2012) [24]
Morocco (Arabs)	30	86.7%	13.3%	
Morocco (Berbers)	66	90.9%	9.1%	

*In vitro* experiments have recently shown that the variant R-allele in *MSTN* rs1805086, which is potentially linked with lower muscle strength and higher obesity risk, is associated with a reduced circulating myostatin activity [13]. The virtual absence of this allele in the Japanese population is in line with that observed in a cohort of healthy young Han Chinese men (frequency of KK genotype of 93.6%) [14], suggesting a highly conserved myostatin activity (that in turn could modulate a shift towards a more oxidative phenotype) [15]. In this regard, we recently postulated that the m.1382A>C polymorphism located in the mtDNA region encoding the recently discovered mitochondrial open reading frame of the 12S rRNA-c (MOTS-c), which is specific for the Northeast Asian population and is associated with metabolic homeostasis and insulin sensitivity, may be among the putative biological mechanisms explaining the high longevity of Japanese people [16].

No differences were found between controls and centenarians in the *ACTN3* R577X allele/genotype distribution. The ‘null’ XX genotype results in complete protein deficiency and the *Actn3*^-/-^ mouse model shows a shift in the properties of fast fibers towards a more oxidative phenotype [17]. Therefore, it might be possible that the X-allele could confer some resistance against metabolism-related diseases and thus contribute, at least partly, to extend life expectancy in some individuals. Previous data in a Spanish cohort showed a similar frequency distribution of the *ACTN3* R577X genotype among centenarians and those humans with the highest oxidative capacity, *i*.*e*., elite endurance athletes [18]. Further, the frequency of the XX genotype in Spanish centenarians was the highest been reported in non-athletic Caucasian populations [18]. Interestingly, the frequency of the XX genotype reported here in male centenarians (n = 117) as well as in supercentenarians of both sexes (110–116 years, n = 82 women and 7 men) are the highest values ever reported for a non-American population [8], *i*.*e*., 33.3% and 32.6%, respectively.

The *ACTN3* XX genotype is present in ~18% of the population of European descent [7], while much lower and higher frequencies have been reported in African and Asian populations, respectively [19, 20]. Such latitude/ethnic heterogeneity has raised the possibility that the X-allele is one of the very few variants associated with gene loss-of-function which have been positively selected over human evolution [7]. This mutation probably preceded the appearance of anatomically modern humans in Europe and Asia (~40.000–60.000 years ago) [8]. Owing to its effect on skeletal muscle metabolism, the X-allele probably provided some sort of functional advantage to modern humans adapting to the novel, colder Eurasian environment that required survival despite scarce food availability.

A main limitation of genetic association studies using a case/control design as the present one is selection of controls. A first potential confounder is differences in date of birth, *e*.*g*., the centenarians and controls of our study were born in the early 1900s and after 1940, respectively. In this regard, the risk of death depends on the interaction of genetic and environmental/lifestyle risk factors; notably, the pattern of exposure to such risk factors is related to year of birth [21]. Second, we cannot infer whether controls will eventually reach the age of 100+ years in the future. In this regard, however, EL remains a rare phenotype worldwide, even in Japan only 25000 centenarians were alive in 2006 [10]. Therefore, the probability of having one potential centenarian in our control cohort is low. In addition, functional studies in model organisms shall be undertaken to unveil the role on EL of the genes that we studied here. We are aware that the cross-sectional nature of our design precludes conclusions on causality. In addition, we focused on EL as a categorical trait (i.e., centenarians versus non-centenarians) and life-span was not considered as a continuous variable (implying that some information could be lost). Finally, caution should be exercised when attempting to extrapolate our findings as being representative to the entire Japanese population. Convenience sampling was used (notably of healthy controls), which is prone to bias (because of population stratification).

In summary, our data showed a virtual absence of the variant (K) allele in *MSTN* rs1805086 in Japanese population, and no differences in allele/genotype frequencies in *ACTN3* rs1815739 among centenarians and healthy controls of this country. More research is needed to unveil the role of these two genes involved in muscle mass conservation and metabolic efficiency, as well as in other gene variants involved in the evolutionary adaptive processes towards a more efficient, oxidative metabolic profile that might characterize the Japanese population, especially its most long-lived individuals.
